# Postmortem Analysis of Optic Nerve Head Vascularization in an Individual With Glaucoma

**DOI:** 10.7759/cureus.59085

**Published:** 2024-04-26

**Authors:** Brianna C Landis, Westin J Wong, Anthony C Pappas

**Affiliations:** 1 Anatomy, Rocky Vista University College of Osteopathic Medicine, Ivins, USA; 2 Gross Anatomy, Rocky Vista University College of Osteopathic Medicine, Ivins, USA

**Keywords:** blood vessel quantification, ocular blood flow, axonal injury, optic nerve head, glaucoma

## Abstract

Reduced ocular perfusion likely contributes to glaucomatous damage at the optic nerve head (ONH). In recent decades, investigators have focused heavily on ocular perfusion pressure and other factors affecting blood flow to the eye. Comparatively, far less attention has been focused on the blood vessels themselves. Here, we asked whether glaucomatous individuals exhibit anatomical deficiencies (i.e., fewer blood vessels) in their ONH blood supply. To answer this question, we performed a systematic literature review to (1) determine how many studies have reported measuring blood vessels in the ONH and (2) whether these studies reported differences in blood vessel quantity. Additionally, we report a method for quantifying blood vessels in ex vivo human ONH preparations, including an ONH from an individual with glaucoma. Our results show that only two studies in the past 50 years have published data concerning blood vessel density in glaucomatous ONHs. Interestingly, both studies reported decreased blood vessel density in glaucoma. Consistent with this finding, we also report reduced blood vessel numbers in the superolateral quadrant of a glaucomatous individual’s ONH. Vascularity in the three remaining quadrants was similar to control. Together, our findings raise the interesting possibility that individuals with a relatively sparse ONH blood supply are more likely to develop glaucoma. Future studies with larger sample sizes and more thorough quantification are necessary to determine the link more accurately between glaucoma and the blood supply to the ONH.

## Introduction

Glaucoma is a leading cause of blindness, affecting 7.7 million people across the globe [1]. Currently, global prevalence is estimated at 3.5% in people aged 40-80 years. However, with a growing proportion of older people in the population, the prevalence of glaucoma is projected to increase substantially in the coming years [2].

The term glaucoma refers to a group of progressive optic neuropathies characterized by excavation of the optic nerve head (ONH), degeneration of retinal ganglion cells (RGCs), and subsequent vision loss [2]. Experimental evidence suggests that (1) injury to RGC axons occurs early in the pathogenesis of glaucoma and (2) the damage is localized to the ONH [3]. The exact nature of this injury, however, is still unknown.

While the pathogenesis of glaucoma has not been fully elucidated, glaucoma is often believed to be primarily a mechanically induced disease [4]. Elevated intraocular pressure (IOP) is considered a key feature of glaucoma and is the result of either oversecretion of aqueous humor from the ciliary body or diminished anterior chamber outflow of aqueous humor through the trabecular meshwork and uveoscleral outflow pathway [4]. Dysregulation of aqueous humor by either mechanism ultimately results in increased IOP, which has been directly correlated to RGC death, glaucoma onset, and progression [5].

Interestingly, a subgroup of glaucoma, termed normal-tension glaucoma, exhibits all the key features of glaucomatous optic neuropathy, including excavation of the ONH, RGC damage, and visual impairment, while maintaining low to normal IOP measurements [6]. Normal-tension glaucoma has challenged the mechanical model of glaucoma to include other potential contributing factors. Recently, other contributing factors such as vascular dysregulation, structural susceptibility of the lamina cribrosa, low intracranial pressure, autoimmunity, and mitochondrial dysfunction have been proposed to be involved in the pathophysiology of glaucoma [7].

Vascular dysfunction has long been considered to play a causal role in the development of glaucoma. Several recent lines of investigation have either described altered ONH hemodynamics in glaucoma, identified low ocular perfusion pressure (OPP) as a risk factor in glaucoma [8-11], or reported reduced ocular blood flow in glaucomatous patients [12-14].

While numerous factors impact blood flow to the ONH, natural anatomical differences in this vascular bed likely exist between individuals and should also be considered (i.e., some people may have slightly more blood vessels in their ONHs, while others have less) [15,16]. In this study, we sought to determine whether glaucomatous ONHs exhibit reduced vascularity in comparison to their healthy counterparts.

To answer this question, we first performed a systematic literature review to identify studies that have quantified blood vessels in the human ONH. Further, we present a novel method for quantifying blood vessels in ex vivo ONHs obtained from human cadaveric donors, including a donor with glaucoma. Together, our findings provide evidence that the blood supply to the ONH is less robust in glaucomatous individuals. This raises the possibility that anatomical inadequacies in the ONH blood supply increase the risk of developing glaucoma, especially when combined with other known risk factors, such as advanced age, or reduced OPP.

## Materials and methods

Systematic literature review

**Figure 1:**
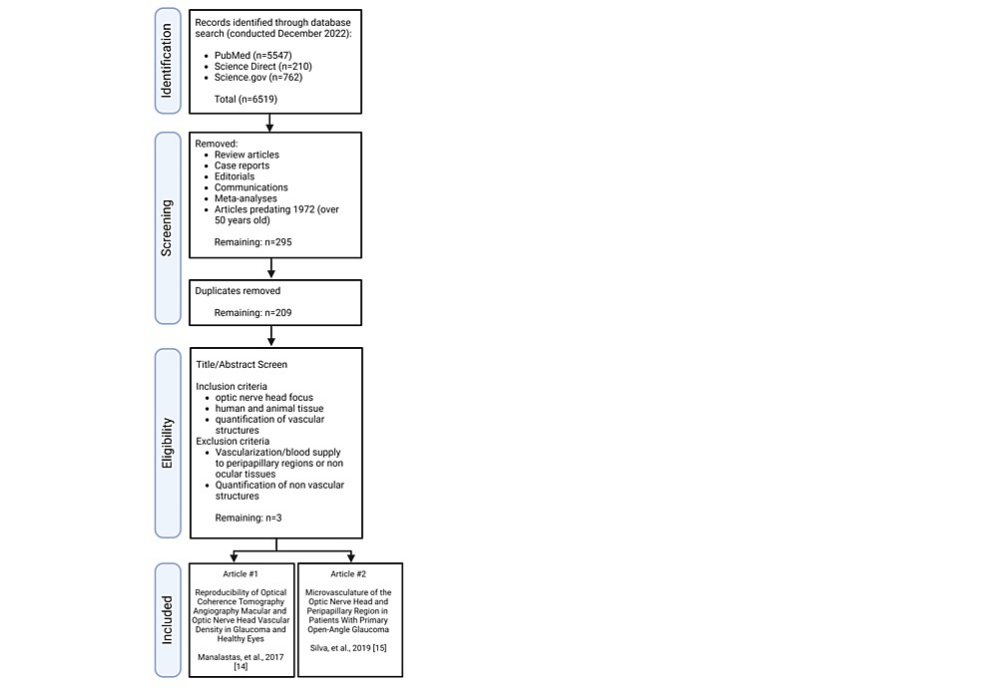
PRISMA flow diagram of systemic literature review search PRISMA, Preferred Reporting Items for Systematic reviews and Meta-Analyses Image created with BioRender.com

The literature was mined from PubMed, Science Direct, and Science.gov search databases using Boolean operator terms: “optic nerve” or “optic nerve head” in conjunction with “quantification”, “vasculature”, “glaucoma”, “blood supply”, and/or “cadaver” (Figure 1). We limited our search to three open-access databases that disseminate peer-reviewed basic science articles and are commonly utilized within the academic community. Original research articles were included, whereas literature reviews, case reports, meta-analyses, editorials, and commentaries were excluded. Inclusion criteria required an ONH focus, human or animal tissue, quantification of vascular structures, research conducted in the setting of glaucoma, and articles published within the past 50 years. Articles that investigated vascularization or blood supply to peripapillary regions or non-optic nerve-associated optic tissues or quantified non-vascular structures were excluded. The primary search was conducted in December 2022.

Quantification of the ONH blood supply

Cadaveric donors were obtained from the University of Utah Body Donation Program. Eyes were removed from the donors, and the ONHs with surrounding sclera were carefully dissected away. Seven ONHs were recovered from five cadaveric donors, of whom one had a history of glaucomatous disease of an unknown subtype. The remaining ONHs were obtained from donors with no history of ophthalmic disease. Cuts were made to the sclera to distinguish anatomical directions (superior, inferior, medial, and lateral). These cuts were later used to orient the image during blood vessel quantification.

Once prepared, isolated ONHs were sent to HistoWiz for sectioning, staining, and imaging. Serial sections (5-µm thick) were collected and stained with an antibody targeting CD31, a protein constitutively expressed by vascular endothelial cells.

The scleral cuts were used to guide the placement of a “crosshair” over the ONH, dividing it into four quadrants. The 10× images were then acquired from each quadrant and used for blood vessel quantification. Images were captured and de-identified by investigator ACP. The images were then shared with investigators BCL and WJW. Vascularity (i.e., the number of CD31-positive blood vessels) was quantified manually by a blinded observer (either BCL or WJW). Six serial sections per ONH were analyzed. CD31-positive structures were included in our analysis if they exhibited either (1) a clear and obvious lumen or (2) 20 µm of uninterrupted staining. The central retinal vessels were excluded from our analysis.

## Results

Our systematic literature review yielded two articles that quantified blood vessels within the ONH (Table 1). Both articles employed optical coherence tomography angiography (OCT-A) to determine ONH blood vessel density in glaucomatous and non-glaucomatous human eyes. The first of these studies was conducted in 2017 and aimed to determine the reproducibility of OCT-A measurements with regard to ONH and macular vessel density [17]. Reproducibility was defined using coefficients of variation (CV) calculated from variance component models. Fifteen healthy volunteers, in addition to 14 glaucoma patients, were enrolled, with results showing good reproducibility of OCT-A ONH and macular vessel density. Interestingly, glaucoma patients were also found to have sparser vessel density (interclass correlation measurements of retinal nerve fiber layer in healthy eyes were 0.65-0.85 compared to glaucomatous eyes 0.89-0.94) and poorer reproducibility than healthy controls (1.8-3.2% CV of vessel density in healthy ONH compared to 2.3-4.1% CV of vessel density in glaucomatous ONH) [17].

**Table 1:** Summary of the two final articles included following the literature review OCT-A, optical coherence tomography angiography; ONH, optic nerve head; POAG, primary open-angle glaucoma

Investigators and year	Species	In vivo or ex vivo	Method	Glaucoma (yes/no)	Study purpose	Conclusion
Manalastas et al. (2017) [17]	Homo sapiens	In vivo	OCT-A	Yes	Determine the reproducibility of ONH and macular vessel density measurements with OCT-A for diagnostic purposes	The reproducibility of OCT-A measurements is good. Glaucoma patients have sparser vessel density and poorer reproducibility than their healthy counterparts.
Nascimento E Silva et al. (2019) [18]	Homo sapiens	In vivo	OCT-A	Yes	Assess ONH and peripapillary microvasculature in POAG using OCT-A	Patients with POAG demonstrated compromised microvasculature in the deep ONH and peripapillary regions compared to the non-POAG control group.

A later study in 2019 again employed OCT-A to compare the ONH and peripapillary microvasculature density in glaucomatous and non-glaucomatous human eyes. OCT-A images from 30 primary open-angle glaucoma (POAG) patients and 16 healthy controls were analyzed [18]. Again, the investigators reported decreased blood vessel density in the ONH when compared to healthy controls: POAG eyes showed significantly lower vessel density (39.4% ± 4.0%) and flow (38.8% ± 5.6%) in deep ONH, peripapillary vessel density (37.9% ± 2.9%), and flow (43.6% ± 4.0%) compared with control eyes (44.1% ± 5.1%, 44.7% ± 6.9%, 40.7% ± 1.7%, and 47.8% ± 2.5%, respectively; P ≤ 0.007 for all) [18].

**Figure 2:**
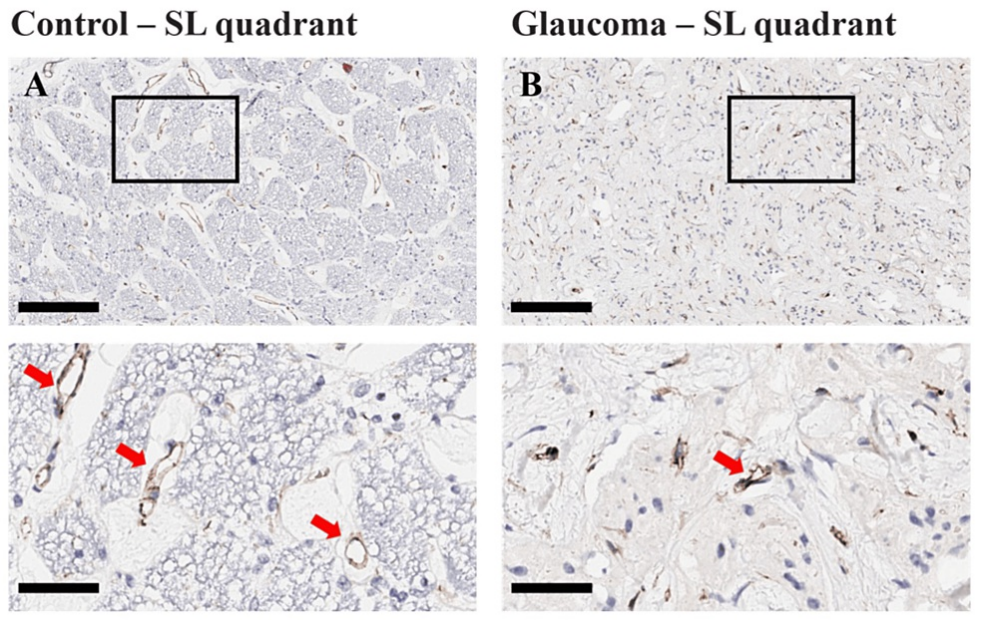
Ex vivo quantification of ONH blood vessels. 10× (top row) and 40× (insets) images showing the SL quadrants of (A) control and (B) glaucomatous ONHs. Blood vessels are indicated by the red arrows (insets only; A, B). Scale bars are 200 µm (10× images) and 50 µm (insets) ONH, optic nerve head; SL, superolateral

Using our novel ex vivo approach, we measured vascularity within the four quadrants of the ONH (IL, inferolateral; IM, inferomedial; SL, superolateral; and SM, superomedial). We found that the SL quadrant of the glaucomatous ONH exhibited a consistently lower number of blood vessels (µ = 16.0, SE = 1.7; n = 6 images from one eye) compared to the SL quadrants of non-glaucomatous ONHs (µ = 27.5, SE = 4.3; n = 24 images from five eyes) (Figure 2).

**Figure 3:**
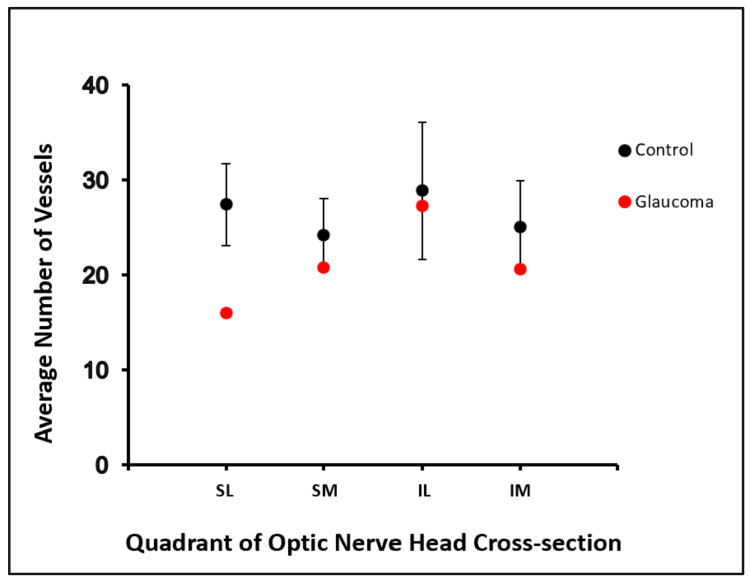
Summary data showing the mean number of vessels within each quadrant of the ONH IL, inferolateral; IM, inferomedial; ONH, optic nerve head; SL, superolateral; SM, superomedial

Vascularity within the SM, IM, and IL quadrants was similar between glaucomatous and non-glaucomatous ONHs (Figure 3). Our results are consistent with the aforementioned in vivo studies reporting sparser blood vessel density in the ONHs of glaucomatous patients.

## Discussion

In this study, we attempted to investigate if glaucomatous ONHs exhibit a reduced quantity of blood vessels. In reviewing the literature, we identified two original research articles that quantified vascular structures in the ONH. In both studies, the investigators directly measured blood vessels in the ONHs of living glaucoma patients using OCT-A. Both studies reported reduced blood vessel density in glaucomatous ONHs compared to healthy controls.

Here, we corroborate these in vivo findings by showing a decreased number of blood vessels within the SL quadrant of an ONH obtained from a cadaveric donor with glaucoma. The reduction in blood vessels could stem from one or more of the following: (1) reduced number of vessels entering the nerve parenchyma within the SL quadrant of the ONH; (2) reduced branching of vessels within the SL quadrant of the ONH; and (3) remodeling and/or paring back of existing vessels due to the loss of axons within the SL quadrant of the ONH.

It is reasonable to assume that natural inter- and intraindividual variations exist in the ONH blood supply. Natural inadequacies in the ONH vasculature may, over time, increase the likelihood of axonal damage and RGC death, especially when combined with other known risk factors for glaucoma [19]. Elevated IOP, for example, decreases OPP, thereby limiting the driving force for the delivery of glucose and oxygen to the ONH [20]. Moreover, IOP-induced mechanical deformation at the ONH could further restrict blood flow by directly pinching off ONH capillaries [21]. Advanced age, another risk factor for glaucoma, has been shown previously to widen perivascular spaces and increase collagen deposits around ONH capillaries [22]. These changes would likely impede the transfer of oxygen and nutrients into the surrounding milieu.

For many years, investigation into the role of vasculature in glaucoma has focused heavily on OPP and other factors affecting blood flow to the eye. Comparatively, far less attention has been focused on the blood vessels themselves. Interestingly, known risk factors for glaucoma, such as smoking and diabetes, are widely accepted to play a role in vascular dysregulation through enhancing apoptotic and degenerative processes triggered by oxidative damage, raising the question of whether the intrinsic properties of dysfunctional blood vessels could contribute to this increased risk of glaucoma [19,23,24]. Moreover, axons within the ONH are unmyelinated, which would increase the metabolic demand for action potential generation [25,26]. Such vascular disturbances set off a destructive chain of events: endothelial dysfunction increases a proinflammatory and prothrombotic state and results in the synthesis of nitric oxide (NO), a free radical, which further triggers cell destruction [19,27]. Genetically, an association between NO and POAG has been reported in the literature [28] as have the toxic effects of NO on RGCs [28-33]. Furthermore, accumulated free radicals have been shown to damage the structure of the trabecular meshwork and cell adhesion and increase outflow resistance [19,34,35] and the ONH has demonstrated similar alterations [36]. While the pattern of this vascular destruction has not been fully elucidated (i.e., reduced vessel number, branching, and remodeling), research into the macular vascular networks of individuals with normal-tension glaucoma revealed reduced branching complexity in those who also had systemic hypertension [37].

Glaucoma patients often suffer from additional systemic vascular disorders, suggesting glaucoma could exist on a spectrum of systemic vascular abnormalities. For example, individuals with glaucoma have been found to exhibit reduced blood flow in other body regions, such as reduced levels of nail-fold capillary flow [38,39]. Other indirect demonstrations of altered blood flow in individuals with glaucoma have been represented through changes seen in conjunctival capillaries [40,41], increased prevalence of ONH hemorrhages [42,43], venous thrombosis [44,45], ischemic brain lesions [46-52], and silent myocardial ischemia. Systemic deficiencies in blood vessel integrity have also been shown in glaucoma patients, who exhibit a higher prevalence of nocturnal hypotension [53,54] and vasospasm and migraine [55-59].

We do not intend to discredit elevated IOP as a major risk factor for glaucoma neuropathy, as it may perhaps be the most important singular factor. However, the pathogenesis of glaucoma is likely multifactorial, and recent extensive investigations have supported that multiple risk factors exist, such as individual variations, vascular disturbances, advanced age, toxic effects, and oxidative damage caused by reactive oxygen species, among others [60].

In experimental glaucoma, early axonal damage is initiated at the dorsal and dorsolateral (DL) regions of the ONH [22,61,62]. These observations fit with the findings in our current study. Here, we consistently detected the fewest blood vessels in the SL quadrant of a glaucomatous ONH. The SL quadrant in bipedal animals is equivalent to the DL quadrant in quadrupeds. It is tempting to think that a natural shortage of blood vessels contributed to the development of glaucoma in our cadaveric donors. However, it is unclear whether vascular insufficiency caused axonal damage and RGC death or whether the ONH vasculature responded to the loss of axons by paring back unnecessary branches within the SL quadrant.

Our limited sample size and incomplete information regarding the donor’s eye disease are major weaknesses of this study. However, the results of both our literature review and ex vivo histology show that glaucoma is associated with deficiencies in the ONH blood supply. Future studies, including three-dimensional reconstructions of the ONH vascular bed, will help determine the extent to which this region is supplied in glaucomatous and non-glaucomatous individuals.

## Conclusions

Our study contributes valuable insights into the role of blood vessel density in the ONH concerning glaucoma. An initial review of the literature revealed the scarcity of knowledge on blood vessel quantity in glaucomatous ONHs and underscored an underexplored aspect of glaucoma research, suggesting that anatomical deficiencies in ONH blood supply may contribute to the disease’s development, possibly resulting from various factors such as reduced vessel branching or remodeling. To further investigate this, we obtained ONH from cadaveric donors with and without glaucoma and used histologic analysis to quantify blood vessels. Through this novel ex vivo approach, we found compelling evidence indicating reduced blood vessel density in the superolateral quadrant of the ONH in individuals with glaucoma.

Furthermore, our findings raise the intriguing possibility that natural variations in ONH vasculature among individuals could influence glaucoma susceptibility, particularly when combined with other known risk factors. However, the causal relationship between vascular insufficiency, axonal damage, and RGC death remains unclear, and larger studies with comprehensive quantification are necessary to establish a more accurate link. Future studies, including three-dimensional reconstructions of the ONH vascular bed, will be crucial in determining the extent of vascular supply in glaucomatous and non-glaucomatous individuals, potentially aiding in the development of targeted interventions and preventive strategies for those at risk of glaucoma.
